# Infusion of propofol with bispectral index monitoring does not reduce the amount of propofol used during transvaginal oocyte retrieval procedure

**DOI:** 10.1038/s41598-023-48611-6

**Published:** 2023-12-06

**Authors:** Sevgi Bilgen, Dilek Erdogan, Sabri Berkem Ökten

**Affiliations:** 1 Department of Anesthesiology, Acibadem Kozyatagi Hospital, Ondokuz Mayıs Mah., Begonya Sokak., No:12 Kadıköy, Istanbul, Turkey; 2 Department of Obstetrics and Gynecology, Acibadem Kozyatagi Hospital, Ondokuz Mayıs Mah., Begonya Sokak., No:12 Kadıköy, Istanbul, Turkey

**Keywords:** Medical research, Risk factors

## Abstract

In our study we aimed to investigate whether the use of bispectral index (BIS) monitoring would decrease total propofol consumption during the transvaginal oocyte retrieval procedure. This was a prospective, randomized, controlled, parallel-group clinical trial. The study was conducted in the operating room, and postoperative recovery room. One hundred and thirty, American Society of Anesthesiologists (ASA) I–II patients, over age 18, undergoing transvaginal oocyte retrieval were included in this study. All patients were administered 2 μg/kg fentanyl, and 2 mg/kg propofol for the induction of anesthesia. The patients were divided into two groups. Patients in the group bolus were given 0.5 mg/kg of propofol when necessary, according to the observer's range of motion. Patients in the group BIS were given 10 mg/kg/h propofol infusion adjusted to keep the BIS value between 40 and 60. The primary outcome was the total dose of propofol administered per patient. The secondary outcomes were the time to reach the value of 5 on the Modified Observer's Assessment of Alertness Sedation Scale (MOASs), the time to reach Post Anesthetic Discharge Scoring System (PADSS) ≥ 9 of the patients, satisfaction of the patient, and the gynecologist. The amount of total propofol was higher in the group BIS than in the group bolus administered according to the patient's clinic. There was no difference in the time to reach the value of 5 on the MOASs between the groups. The time to reach PADSS ≥ 9 was longer in the group BIS than in the group bolus. There was no difference between the two groups in terms of the satisfaction of the patient and the gynecologist. Administration of propofol as an infusion with BIS monitoring did not reduce the amount of propofol administered to patients during transvaginal oocyte retrieval.

**Clinical trial registration number:** NCT05631925—30/11/2022.

## Introduction

Ultrasound guided transvaginal oocyte retrieval for in vitro fertilization (IVF) is a very common procedure^1^. Although some patients prefer the procedure to be performed without anesthesia, it is recommended to be performed under anesthesia as it is a short but painful procedure^2,3^. Performing the procedure with anesthesia not only increases the comfort of the patient, but also facilitates the procedure, and prevents the patient from being damaged due to trauma during the procedure^3^. For this purpose, different anesthetic techniques such as monitored anesthesia care, sedation, local anesthesia, regional anesthesia, and general anesthesia can be used during the oocyte retrieval procedure^3–5^. The combination of sedative and analgesic drugs with a rapid onset and a short duration of action is ideal in this day-case procedures.

Propofol (2,6-diisopropylphenol) and fentanyl are commonly used agents in oocyte retrieval procedures. Propofol is used as continuous intravenous infusion or intermittent bolus injections^6^.

Although propofol is widely used during oocyte retrieval, studies on its effects on fertilization are controversial. In vitro studies on mouse oocytes^6,7^ and some human studies have shown that propofol may be dose- and time-dependent detrimental to fertilization^8^. According to the results of a retrospective cohort study examining the effects of general anesthesia with propofol during oocyte retrieval on oocyte fertilization, embryo development and implantation, general anesthesia causes a significantly lower oocyte fertilization rate^9^. Propofol is detected in significant concentrations in the follicular fluid, depending on the dose and the duration of propofol administration^10^. Due to its potential adverse effects on fertilization, there are studies suggesting that propofol should be used with caution during oocyte retrieval^6^ the total dose should be strictly limited^10^. Therefore, our aim is to use as low amount of propofol as possible during oocyte retrieval.

BIS monitoring systems provide an objective measure of a patient's depth of consciousness^11^. Numerical values of BIS are between 0 and 100. The range 0–40 clinically indicates deep anesthesia and burst suppression. 60–70 indicates recovery from general anesthesia and light anesthesia, and 70–100 indicates being awake^12^. A BIS value between 40 and 60 indicates the appropriate level of general anesthesia recommended by the manufacturer and in previous studies^13,14^. It has been demonstrated that titration of propofol with BIS monitoring during anesthesia reduces propofol use^11,15^.

Our hypothesis was that during the transvaginal oocyte retrieval procedure, the amount of propofol given as an infusion with BIS monitoring would be less compared to the amount used when bolus doses are given according to the clinical evaluation.

## Materials and methods

This was a prospective, randomized, parallel-group clinical trial. This study was conducted between 27 May 2022 and 17 August 2022 in the IVF unit. The protocol was registered on the http://www.clinicaltrials.gov protocol registration system (NCT05631925—30/11/2022) and was approved by the Clinical Research Ethics Committee of Acıbadem University, Istanbul, Turkey (No:2022/05-32). All methods were performed in accordance with relevant guidelines and regulations.

One hundred and thirty American Society of Anesthesiologist’s Class I or II patients, over age 18, undergoing transvaginal oocyte retrieval for in vitro fertilization under general anesthesia were included in this study. Patients were excluded if they had contraindication for general anesthesia, they had a history of mental illness, alcohol, or substance abuse. Patients were randomized into two groups (65 patients in group bolus, 65 patients in group BIS) by an anesthesiologist who was not included in the study. Randomization was performed by opening one of a series of sequentially numbered opaque envelopes that contained the group assignment. Written informed consent was obtained from all study participants. The patients, and the gynecologist were blinded to group assignment.

Each patient’s age, weight, height, body mass index (BMI), and ASA classification were recorded preoperatively. All the patients fasted for at least 6 h prior the procedure and did not receive premedication. On the arrival in the operating room, heart rate, noninvasive blood pressure, and oxygen saturation on pulse oximetry were monitored, an intravenous canula was inserted and 0.9% sodium chloride (NaCl) infusion was started. Patients in group BIS were also monitored with BIS.

In both groups, anesthesia was induced with intravenous propofol (2 mg/kg) and fentanyl (2 μg/kg) administration. A second-generation laryngeal mask was inserted two minutes after the anesthesia induction in group bolus and when BIS value < 60 in group BIS. Normocapnic mechanical ventilation was initiated. A 40% oxygen/air mixture was used during the procedure. Laryngeal mask placement was attempted 2 times, if unsuccessful, ventilation with face mask was continued, and the patient was excluded from the study. The number of insertion attempts was recorded.

In group bolus, 0.5 mg/kg propofol was administered when the intraoperative movement scale (IOMs) was ≥ 2.

### Intraoperative movements Scale (IOMs)^13^

Grade 0: = No movement.
Grade 1: = Ankles movement (feet dorso-flexion).
  Non procedure interferent.
  It could deepen analgesia.
Grade 2: = Knee movements (legs flexo-extension) (with/without movements).
  Non procedure interference (aspiration could stop).
  It could deepen analgesia/anesthesia.
Grade 3: = Pelvis/ hips movements (with/without legs/thighs movements).
  Aspiration must be stopped.
  Must be deepen analgesia/anesthesia.
Grade 4: = Rude movements of the pelvis, chest and/or arm.
  Aspiration must be stopped.
  Must be deepen anesthesia.

In group BIS continuous propofol infusion (10 mg/kg/h) was started immediately after anesthesia induction^10^. In these patients, the propofol infusion rate was adjusted to keep the BIS value between 40 and 60 during the procedure. If the BIS value was above the target level, the propofol infusion rate was increased by 20%, and if the BIS was below the target level, the infusion rate was decreased by 20%. Propofol infusion was stopped when oocyte aspiration was completed.

During the procedure, all the patients received paracetamol (1 g) intravenously, and diclofenac sodium 100 mg suppository was applied at the end of the procedure for postoperative analgesia. The laryngeal mask was removed when the patients opened the eyes.

Heart rate, oxygen saturation, systolic, diastolic, and mean blood pressure were recorded before anesthesia, at the 5th, 10th, and 15^th^ mins of the procedure, at the end of the procedure and when the patient was awake. BIS values were also recorded in the group BIS at the same time points. Bradycardia (defined as heart rate < 25% from baseline or < 50 beats/min), hypotension (defined as systolic blood pressure < 25% from baseline or < 90 mmHg), and presence of rigidity were recorded. In the presence of bradycardia, 0.5 mg atropine was administered. In the presence of hypotension, 5 mg ephedrine was administered.

The duration of anesthesia and duration of procedure were recorded. The total amount of propofol was recorded. After the removal of the laryngeal mask, the time to reach 5 in the MOASs was recorded in the operating room.

### Responsiveness scores of the modified observer’s assessment of alertness/sedation scale (MOASs)^16^

**Table:** 

Response	Score level
Responds readily to name spoken in normal tone	5 (alert)
Lethargic response to name spoken in normal tone	4
Responds only after name is called loudly or repeatedly	3
Responds only after mild prodding or shaking	2
Does not respond to mild prodding or shaking	1

All the procedures were performed by same experienced gynecologist and after the procedure, the satisfaction with the anesthesia was questioned. Gynecologist satisfaction 1: very satisfied 2: satisfied 3: not satisfied 4: not satisfied at all.

After transfer to the recovery area, patients were assessed continuously by a nurse who was blinded to the group assignment. Each patient’s pain was evaluated using visual analogue scale (VAS) ranging from 0 cm (no pain) to 10 cm (worst pain imaginable) during the postoperative period. If the patient had a VAS score ≥ 4, 75 mg diclofenac sodium was administered intramuscularly and recorded as additional analgesic.

Nausea and vomiting of the patients were evaluated with a 4-grade scale 0: no nausea or vomiting 1: tolerable nausea or simple vomiting that does not require treatment 2: intolerable nausea or recurrent vomiting requiring treatment 3: recurrent nausea or vomiting resistant to drug therapy. If the score ≥ 2 ondansetron 4 mg was administered intravenously. Nausea and vomiting score and the use of antiemetics were also recorded.

The time of patients to reach PADSS ≥ 9 was recorded.

### Post anesthetic discharge scoring system (PADSS)^16^

**Table:** 

Vital signs	2 = Blood pressure and heart rate within 20% of preoperative value
	1 = Blood pressure and heart rate within 20–40% of preoperative value
	0 = Blood pressure and heart rate more than 40% different from the preoperative value
Activity and mental status	2 = Oriented, and has a steady gait
	1 = Oriented or has a steady gait
	0 = Neither
Nausea and or vomiting	2 = Minimal
	1 = Moderate, having required treatment
	0 = Severe, requiring treatment
Pain	2 = Minimal
	1 = Moderate, having required treatment
	0 = Severe, requiring treatment
Surgical bleeding	2 = Minimal
	1 = Moderate
	0 = Severe

Patient’s satisfaction related to the anesthetic method was questioned before discharge. Patient satisfaction 1: very satisfied 2: satisfied 3: not satisfied 4: not satisfied at all (I had pain during the procedure, I was awake during the procedure). The score was recorded.

The primary outcome of our study was the total dose of propofol administered to the patients. The secondary outcomes were the time to reach the value of 5 on the MOASs, the time to reach the value of PADSS ≥ 9 in the postoperative period, and the satisfaction of the patient and the gynecologist.

### Statistical analysis

The primary outcome of the study was total propofol consumption. The power analysis was based on the results of the study conducted by Luginbuhl et al.^15^. The authors found that total consumption of propofol was 6.03 ± 1.4 mg/kg and 6.64 ± 0.9 mg/kg consecutively in groups using BIS and not using BIS (p = 0.023). With a power of 80% and an alpha error of 5%, the sample size calculation determined that 60 patients were required for each group, using the G * Power (v3.1.7) program. Considering the possibility of drop out and lack of data, the total number of patients required for the study was determined to be 130 (65 patients for each group). We analyzed the data with SPSS version 16 (SPSS Inc., Chicago, Illinois, USA). Convenience of parameters to the normal distribution was assessed with Shapiro Wilks test while assessing the study data. Student t-test was used in comparing quantitative data for comparing parameters that showed normal distribution between the two groups, while Mann–Whitney *U* test was used in comparing data that did not show a normal distribution between the two groups. Chi-square test and Fisher’s exact tests were used in comparing qualitative parameters. p < 0.05 was considered statistically significant.

### IRB number

The Clinical Research Ethics Committee of Acıbadem University, Istanbul, Turkey (No:2022/05-32).

## Results

One hundred and thirty patients were enrolled in the study, eight patients were excluded due to deviation from the protocol. Two of the patients in group bolus had paracetamol allergy. In one patient from the same group, the laryngeal mask could not be placed despite two attempts because the mouth opening was not sufficient, and face mask ventilation was performed. One patient in this group had excessive rigidity. It was necessary to administer muscle relaxant and the patient was excluded from the study. Three of the patients in group BIS required a bolus of propofol after the infusion was stopped, because the bleeding control was longer than expected. One patient had a BIS value of 80 after placement of a laryngeal mask, and bolus propofol was required to provide adequate anesthesia despite increasing the infusion dose. As a result, one hundred and twenty-two patients completed the study: group bolus, n = 61 and, group BIS, n = 61.

The age, weight, height, BMI, and the distribution of ASA score were similar in both groups (Table 1).

**Table 1:** Demographic characteristics, number of attempts for laryngeal mask insertion, the patient, and the gynecologist satisfaction, bradycardia, hypotension, and rigidity rate in the groups.

	Group bolus (n = 61), mean ± SD	Group BIS (n = 61), mean ± SD	*P* value
Age (year)	37.16 ± 5.90	37.39 ± 5.98	0.832^1^
Weight (kg)	63.42 ± 9.79	64.91 ± 10.88	0.428^1^
Height (cm)	163.32 ± 6.17	162.78 ± 6.33	0.634^1^
BMI (kg/m^2^)	23.49 ± 3.63	24.21 ± 4.63	0.341^1^
ASA I/ASA II	51/10	43/18	0.131^3^
Laryngeal mask placement 1 attempt/2 attempts	58/3	56/5	0.717^3^
Patient satisfaction: 1/2/3/4	59/2/0/0	55/6/0/0	0.272^3^
Gynecologist satisfaction: 1/2/3/4	54/5/1/1	58/3/0/0	0.450^2^
Bradycardia	11 (%18)	27 (%44)	*0.003^3^
Hypotension	15 (%24)	22 (%36)	0.237^3^
Rigidity	1 (%0.01)	1 (%0.01)	1^3^

*Group bolus:* Propofol bolus group, *Group BIS:* Propofol infusion group, *ASA:* American Society of Anesthesiologist. *Statistically significant (p < 0.05).

^1^Student’s t-test, ^2^Chi-square test, ^3^Fisher’s Exact test.

There was no difference between the groups regarding of number of attempts for the insertion of the laryngeal mask (Table 1).

No hemodynamic difference was observed between the groups, except the occurrence of bradycardia during the procedure. More patients in group BIS had bradycardia than patients in group bolus, therefore, the number of patients treated with atropine was different between the groups: group BIS (27 patient) vs group bolus (11 patient), p = 0.003 (Table 1). Rigidity was observed in one patient in each group (p = 1) (Table 1).

**Figure 1:**
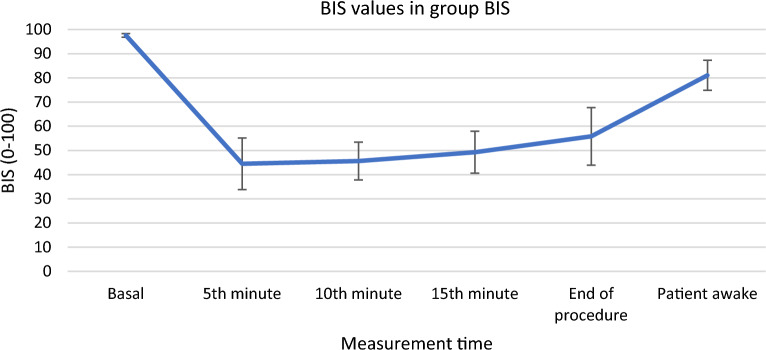
BIS values in group BIS. *BIS:* Bispektral index, values are given as mean ± standard deviation.

Figure 1 shows the group BIS values before anesthesia, at the 5th, 10th, and 15th minutes, at the end of the procedure and when the patients are awake.

There was no difference between groups in terms of duration of anesthesia (22.37 ± 6.79 min vs 23.04 ± 8.09 min) and duration of procedure (16.63 ± 6.62 min vs 16.19 ± 7.61 min) (respectively, p = 0.620, p = 0.733) (Table 2).

**Table 2:** Duration of anesthesia, and procedure, duration to reach 5 on the MOASs, duration to reach PADSS ≥ 9 (min), total amount of propofol.

	Group bolus (n = 61), mean ± SD	Group BIS (n = 61), mean ± SD	*P* value
Duration of anesthesia (min)	22.37 ± 6.79	23.04 ± 8.09	0.620^1^
Duration of procedure (min)	16.63 ± 6.62	16.19 ± 7.61	0.733^1^
Duration to reach 5 on the (MOASs) (min)	0.96 ± 1.09	0.80 ± 0.87	0.362^1^
Duration to reach PADSS ≥ 9 (min)	71.72 ± 25.41	83.69 ± 32.40	*0.026^1^
Total amount of propofol (mg)	231.06 ± 62.40	278.95 ± 95.04	*0.001^1^

*Group bolus:* Propofol bolus group, *Group BIS:* Propofol infusion group, *MOASs:* Modified Observer’s Assessment of Alertness/sedation scale, *PADSS:* Post Anesthetic Discharge Scoring System; *Statistically significant (p < 0.05); ^1^Student’s t-test.

There was no difference in the time to reach the value of 5 on the MOASs between the groups (Table 2).

The amount of total propofol was significantly higher in the group BIS (278.95 ± 95.04 mg) than in the group bolus (231.06 ± 62.40 mg) (p = 0.001) (Table 2).

In group bolus, an average of 3.26 ± 2.01 bolus propofol was required during the procedure after anesthesia induction. In group BIS, propofol infusion was adjusted an average of 2.55 ± 2.20 times during the procedure. In 9 of the patients in group BIS, the propofol infusion rate was stable throughout the procedure. The infusion rate was reduced in 32 patients and increased in 9 patients. In 7 patients, the infusion rate was initially reduced and then increased. In 4 patients, the infusion rate was initially increased and then decreased.

The time for the patient to reach the value of PADSS ≥ 9 in the postoperative period was statistically longer in the group BIS (83.69 ± 32.40 min) than in the group bolus (71.72 ± 25.41 min) (p = 0.026) (Table 2).

In the postoperative period, none of the patients needed rescue analgesics. One patient in the group bolus required ondansetron. No patient in the group BIS required antiemetic treatment.

Satisfaction of the patients and the gynecologist did not differ significantly between the groups (Table 1).

## Discussion

In our study, we observed that the amount of propofol administered to patients during the transvaginal oocyte retrieval procedure was higher when administered as an infusion with BIS monitoring, compared to the administration as bolus doses according to the clinical conditions of the patients.

There are studies showing that the success of the oocyte retrieval process does not change with the applied anesthesia methods^5,17,18^. But there is increasing concern about the potential detrimental effects of different types of anesthesia on the quality of the oocytes and, consequently, on the reproductive outcome^3^. Since it has been shown that exposure to anesthetic drugs, especially for a long time, has negative effects on fertilization^10,19^, it is recommended that the oocyte should be exposed to anesthetic agents at a minimum level^19,20^. Therefore, it is critical to administer the appropriate agent at the appropriate dose and duration in anesthesia^21^.

Propofol is one of the most preferred drugs for anesthesia in oocyte retrieval, due to its short onset time and rapid recovery from anesthesia^16^. Nevertheless, there are conflicting results regarding the effect of propofol used in oocyte retrieval on fertilization. In their 1997 editorial, Hein and his colleagues ask the following question: "What we do really know about propofol's effects on human reproduction?"^22^. It seems very difficult to find the exact answer to this question. The results of studies are confusing.

Alsalili et al., in their study on the effect of propofol on oocyte maturation and fertilization, concluded that although propofol did not affect fertilization, high propofol concentrations could impair in vitro oocyte maturation in mice^23^. Although Ben-Shlomo et al. could not show a relationship between the duration of anesthesia and propofol concentrations in the follicular fluid^24^, Janssenwillen et al. conducted a study on mouse oocytes and showed that propofol accumulates in the follicular fluid in a dose- and time-dependent manner^6^. They suggested caution in the clinical use of propofol during oocyte retrieval^6^. In the study conducted by Budak et al. during the IVF process for transvaginal oocyte collection in rats, it was shown that the number of embryos, their quality and the number of offspring decreased as the exposure time to propofol increased^25^. Similarly, to animal studies, it was determined that propofol accumulates in the follicular fluid depending on the dose and duration in studies conducted in humans^8,10^. In another randomized controlled study, it was found that although there was no significant difference in fertilization rates between high- and low-dose propofol, high-dose propofol administration had a negative effect on human embryo development in the late period and caused a decrease in the clinical pregnancy rate^26^. Considering the possible adverse effects on fertility during anesthesia, it is recommended to limit the total dose and duration of propofol administration^8,10^.

In various studies comparing the use of propofol administered as a continuous infusion with an intermittent bolus during sedation or general anesthesia, and in a meta-analysis covering 12 studies, it was found that propofol was used in higher doses when given as an infusion compared to the bolus dose, without causing an increase in adverse effects^27–29^. However, in previous studies using propofol infusion, it has been demonstrated that using BIS monitoring reduces the amount of anesthetic drug used^11,15^. It was also stated in these studies that the recovery of patients after anesthesia was faster^11,15^.

Gan et al. demonstrated that adding BIS monitoring to standard anesthesia practice reduced the propofol infusion rate and the total amount of propofol used. In our study, we could not observe similar results in general anesthesia applied with BIS monitoring. In their study, Gan et al. adjusted their propofol infusions to reach the target BIS value of 45–60, but they allowed the BIS value to increase to 60–75 in the last 15 min of the case. Considering that our target value for BIS value is in the range of 40–60 and the average procedure time is 16 min in our study, it is understood that the target BIS values are very different from the study of Gan et al.^11^. They also showed that BIS facilitates the titration of propofol, which improves patients' recovery from anesthesia^11^. In the postoperative period, the time to reach the PADSS ≥ 9 value, which was considered suitable for discharge, was longer in the group BIS than in the group bolus in our study. This result is not surprising considering that the total dose of propofol used was higher in the group BIS than in the bolus group.

Luginbühl et al. reported that BIS monitoring reduced propofol use and accelerated recovery after propofol anesthesia. In this study, it was stated that the drug concentration was adjusted to keep the BIS between 45 and 55 during the surgery in patients with BIS monitoring. However, if the BIS increased above the target level, they preferred to apply muscle relaxants before increasing the drug concentration^15^. It is not surprising that the amount of propofol used with this method is low. In our study, the highest targeted BIS value was 60 during the procedure, and propofol infusion rate was increased instead of giving muscle relaxants when the BIS value of the patients exceeded the target value. In addition, in the above-mentioned study, the BIS value was allowed to rise up to 65 in the last 15 min of the procedure. In this case, it can be said that the target BIS value in our study is completely different from this study. Therefore, it was inevitable that we would come to a different conclusion from Luginbuhl et al.^15^.

Based on the results of our study, is it realistic to say that the amount of propofol used increased by using BIS, contrary to the results of previous studies? Or should a more appropriate target be determined for BIS value during anesthesia for oocyte retrieval? Circeo et al. observed that the mean BIS ranged from 47 to 53 during oocyte retrieval, and they recommended this range as a target. However, artificial airway insertion was not used in any patient in this study^1^. Since we are used to insert laryngeal masks under general anesthesia in all patients during oocyte retrieval, we targeted the BIS value within the recommended range of 40–60 for general anesthesia.

In the present study, the patients in the bolus group were given propofol according to their clinical responses, without BIS monitoring. Patients were questioned about their satisfaction before being discharged after the procedure. Fifty-nine patients in this group said that they were very satisfied with the anesthesia, while the other two patients said they were satisfied. No patient was dissatisfied with the anesthesia method applied. This finding shows that even if propofol is given according to the clinical response of the patient without BIS monitoring, patient satisfaction is high and sufficient depth of anesthesia can be provided for the patient.

We did not use BIS in the group bolus, so we cannot recommend the BIS value that should be targeted for oocyte retrieval based on the results of our study. Considering these findings, we think that the targeted BIS value should be reconsidered for patients undergoing oocyte retrieval under general anesthesia.

In the present study, we could not confirm our hypothesis that we could reduce the amount of propofol administered for general anesthesia in patients undergoing transvaginal oocyte retrieval procedure for IVF treatment, when BIS is targeted in the 40–60 range. This brought us to the conclusion that further studies with more specific methodology and larger series are needed to determine the appropriate target value in the depth of anesthesia when BIS is used during anesthesia for oocyte retrieval.

Intraoperatively, we were unable to use a double-blind study design. The anesthesiologist who administered the anesthesia and recorded the results during the procedure was not blind. This is a major limitation of our study.

### Supplementary Information

Supplementary Information.

## Data Availability

The data was anonymized and stored according to the guidelines of Acıbadem University. The datasets analyzed during the current study are available from the corresponding author on reasonable request.
